# Supplementary figures and images for: Application of the anatomical fiducials framework to a clinical dataset of patients with Parkinson’s disease
Source: Brain Struct Funct. 2021 Oct 23;227(1):393–405. doi: 10.1007/s00429-021-02408-3 (PMC8741686; doi:10.1007/s00429-021-02408-3)

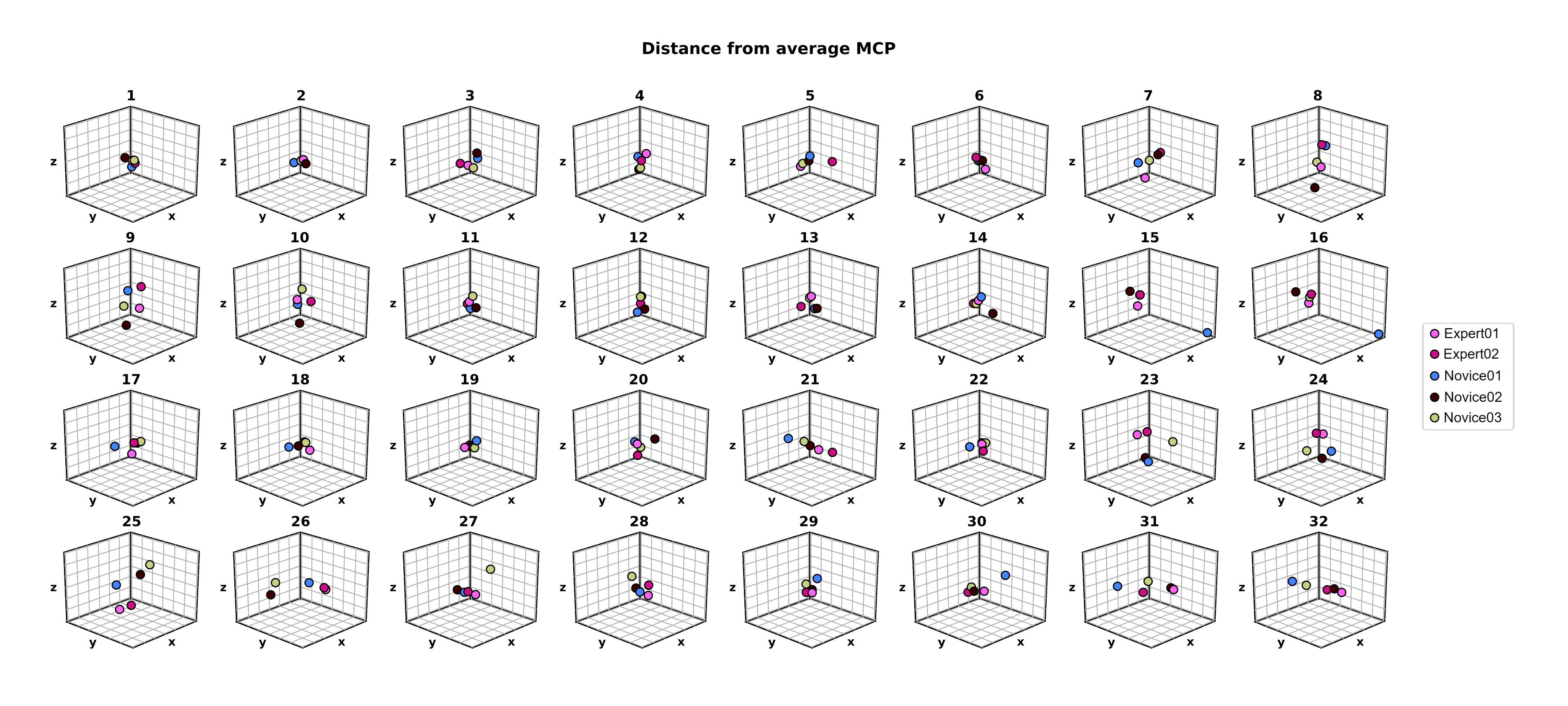

Supplement: Supplementary file 2 — Supplementary file2 (TIF 1418 KB) Online Resource 2 - Mean individual rater Euclidean distance from mid-commissural point (MCP) for all anatomical fiducials in subject space [file 429_2021_2408_MOESM2_ESM.tif]
