# Supplementary material for: Application of the anatomical fiducials framework to a clinical dataset of patients with Parkinson’s disease
Source: Brain Struct Funct. 2021 Oct 23;227(1):393–405. doi: 10.1007/s00429-021-02408-3 (PMC8741686; doi:10.1007/s00429-021-02408-3)
Supplement: Supplementary file 3 — Supplementary file3 (PDF 91 KB) Online Resource 3 - Mean real-world and consensus anatomical fiducial registration error (AFRE) with standard deviation obtained with linear and non-linear registration of clinical images to MNI space using fMRIPrep [file 429_2021_2408_MOESM3_ESM.pdf]

Fiducial	Fiducial Name	Real-World Linear AFRE (mm)	Real-World Non-linear AFRE (mm)	Consensus Linear AFRE (mm)	Consensus Non-linear AFRE (mm)
1*†	AC	2.39 ± 1.34	1.11 ± 1.06	2.17 ± 1.20	0.78 ± 0.59
2	PC	2.43 ± 1.12	2.05 ± 1.27	2.34 ± 1.08	1.95 ± 1.25
3	Infracollicular sulcus	2.60 ± 1.49	2.12 ± 1.56	2.30 ± 1.30	1.69 ± 1.32
4*	PMJ	4.49 ± 1.79	2.82 ± 1.36	4.40 ± 1.63	2.63 ± 1.16
5*†	Superior interpeduncular fossa	2.61 ± 1.10	2.06 ± 1.12	2.12 ± 0.88	1.44 ± 0.89
6	R superior LMS	2.80 ± 1.36	3.10 ± 1.50	2.37 ± 0.85	2.66 ± 1.20
7	L superior LMS	2.82 ± 1.43	3.21 ± 1.70	2.30 ± 0.95	2.76 ± 1.40
8†	R inferior LMS	3.11 ± 1.54	2.86 ± 1.65	2.30 ± 1.05	1.89 ± 1.04
9†	L inferior LMS	3.36 ± 1.54	3.01 ± 1.82	2.56 ± 1.19	2.20 ± 1.15
10	Culmen	3.83 ± 1.74	4.32 ± 1.98	3.21 ± 1.66	3.76 ± 1.74
11	Intermammillary sulcus	2.68 ± 1.51	2.05 ± 1.09	2.56 ± 1.43	1.86 ± 1.03
12	R MB	2.83 ± 1.53	2.02 ± 1.05	2.68 ± 1.43	1.80 ± 0.97
13	L MB	2.89 ± 1.55	2.14 ± 1.11	2.74 ± 1.45	1.95 ± 0.99
14	Pineal gland	2.88 ± 1.33	3.45 ± 1.74	2.33 ± 1.12	3.12 ± 1.28
15*	R LV at AC	5.48 ± 3.59	3.36 ± 3.28	4.93 ± 2.27	2.67 ± 1.67
16*	L LV at AC	5.71 ± 3.64	3.16 ± 3.49	4.97 ± 2.54	2.51 ± 1.85
17*	R LV at PC	4.72 ± 2.71	3.21 ± 2.47	4.23 ± 2.23	2.69 ± 1.91
18*	L LV at PC	4.86 ± 2.63	2.96 ± 2.10	4.49 ± 2.56	2.49 ± 1.93
19*	Genu of CC	3.81 ± 1.90	2.51 ± 1.35	3.50 ± 1.86	2.25 ± 1.06
20	Splenium	3.17 ± 1.67	3.39 ± 1.13	2.80 ± 1.46	3.25 ± 0.84
21*	R AL temporal horn	4.43 ± 1.53	2.60 ± 1.72	3.89 ± 1.45	1.92 ± 1.09
22*	L AL temporal horn	5.09 ± 2.00	3.26 ± 2.38	4.48 ± 1.71	2.39 ± 1.74
23	R superior AM temporal horn	4.24 ± 2.27	4.66 ± 2.17	3.86 ± 1.64	4.42 ± 1.53
24	L superior AM temporal horn	5.30 ± 2.67	5.14 ± 2.24	4.96 ± 1.96	4.82 ± 1.63
25*	R inferior AM temporal horn	5.94 ± 2.92	4.41 ± 2.71	5.26 ± 1.96	3.27 ± 1.71
26	L inferior AM temporal horn	6.28 ± 3.39	4.81 ± 3.30	5.70 ± 2.04	3.84 ± 2.07
27*	R indusium griseum origin	4.50 ± 1.79	3.50 ± 1.91	3.95 ± 1.47	2.82 ± 1.49
28*	L indusium griseum origin	5.35 ± 2.15	4.20 ± 1.80	4.95 ± 1.69	3.64 ± 1.33
29	R ventral occipital horn	7.43 ± 2.89	6.81 ± 2.94	6.99 ± 2.83	6.54 ± 2.42
30	L ventral occipital horn	7.42 ± 3.18	7.36 ± 3.41	6.89 ± 3.25	6.86 ± 3.48
31*†	R olfactory sulcal fundus	3.52 ± 1.92	2.60 ± 1.89	2.66 ± 1.35	1.77 ± 0.89
32*†	L olfactory sulcal fundus	3.84 ± 1.89	2.58 ± 1.85	3.06 ± 1.37	1.58 ± 0.83
**Mean**		**4.15 ± 2.03**	**3.34 ± 1.94**	**3.69 ± 2.20**	**2.82 ± 2.01**

Online Resource 3 – Mean real-world and consensus anatomical fiducial registration error (AFRE) with standard deviation obtained with linear and non-linear registration of clinical images to MNI space using fMRIPrep. Wilcoxon rank-sum tests were obtained for each anatomical fiducial (AFID) between linear and non-linear real-world AFRE, with a significance threshold of 0.05/32 (*). Linear AFRE was significantly greater in 15 AFIDs. Wilcoxon rank-sum tests were obtained for each AFID between non-linear real-world and consensus AFRE, with a significance threshold of 0.05/32 (†). Real-world AFRE was significantly greater in 6 AFIDs. AC, anterior commissure; AL, anterolateral; AM, anteromedial; CC, corpus callosum; IPF, interpeduncular fossa; MB, mammillary body; LMS, lateral mesencephalic sulcus; LV, lateral ventricle; PC, posterior commissure; PMJ, pontomesenphalic junction.
